# A systematic review and meta-analysis of radiotherapy and particle beam therapy for skull base chondrosarcoma: TRP-chondrosarcoma 2024

**DOI:** 10.3389/fonc.2024.1380716

**Published:** 2024-03-19

**Authors:** Masatoshi Nakamura, Masashi Mizumoto, Takashi Saito, Shosei Shimizu, Yinuo Li, Yoshiko Oshiro, Masako Inaba, Sho Hosaka, Hiroko Fukushima, Ryoko Suzuki, Takashi Iizumi, Kei Nakai, Kazushi Maruo, Hideyuki Sakurai

**Affiliations:** ^1^ Department of Radiation Oncology, University of Tsukuba, Tsukuba, Japan; ^2^ Department of Pediatric Radiation Therapy Center/Pediatric Proton Beam Therapy Center, Hebei Yizhou Cancer Hospital, Zhuozhou, China; ^3^ Department of Radiation Oncology, Tsukuba Medical Center Hospital, Tsukuba, Japan; ^4^ Department of Pediatrics, University of Tsukuba Hospital, Tsukuba, Japan; ^5^ Department of Child Health, Institute of Medicine, University of Tsukuba, Tsukuba, Japan; ^6^ Department of Biostatistics, Institute of Medicine, University of Tsukuba, Tsukuba, Japan

**Keywords:** chondrosarcoma, skull base, proton, meta-analysis, systematic review, temporal lobe necrosis, TRP

## Abstract

**Introduction:**

Chondrosarcoma is a rare malignant bone tumor. Particle beam therapy (PT) can concentrate doses to targets while reducing adverse events. A meta-analysis based on a literature review was performed to examine the efficacy of PT and photon radiotherapy for skull base chondrosarcoma.

**Methods:**

The meta-analysis was conducted using 21 articles published from 1990 to 2022.

**Results:**

After PT, the 3- and 5-year overall survival (OS) rates were 94.1% (95% confidence interval [CI]: 91.0-96.2%) and 93.9% (95% CI: 90.6-96.1%), respectively, and the 3- and 5-year local control rates were 95.4% (95% CI: 92.0-97.4%) and 90.1% (95% CI: 76.8-96.0%), respectively. Meta-regression analysis revealed a significant association of PT with a superior 5-year OS rate compared to three-dimensional conformal radiotherapy (p < 0.001). In the studies used in the meta-analysis, the major adverse event of grade 2 or higher was temporal lobe necrosis (incidence 1-18%, median 7%).

**Conclusion:**

PT for skull base chondrosarcoma had a good outcome and may be a valuable option among radiotherapy modalities. However, high-dose postoperative irradiation of skull base chondrosarcoma can cause adverse events such as temporal lobe necrosis.

## 1 Introduction

Chondrosarcoma is a rare malignant bone tumor that originates from cells that produce cartilage (1). It is the second most prevalent sarcoma of the bone, after osteosarcoma (1). Chondrosarcomas can develop anywhere in the body. The most common type found in the skull base is mesenchymal chondrosarcoma, which is typically a malignant tumor with slow growth and a low rate of metastasis (2). However, skull base chondrosarcomas are locally invasive tumors that often pose challenges in achieving complete removal through surgical resection (3). There is also a high incidence of local recurrence, and this has been suggested to be significantly associated with an elevated risk of metastasis and death due to the tumor (3). Therefore, postoperative radiotherapy is generally recommended following as extensive surgical resection as possible (4).

Advances in technology have permitted the use of stereotactic radiotherapy and intensity-modulated radiotherapy for the administration of high doses of radiation to lesions (5). Proton beam therapy (PBT) and carbon-ion radiotherapy (CIRT) are types of particle beam therapy (PT) that offer physical advantages enabling the delivery of a high dose to the target area while minimizing exposure to healthy surrounding organs (6, 7). Good survival and local control rates have also been reported for CIRT in comparison to PBT and definitive irradiation of unresectable cases (7). Since skull base chondrosarcoma is a rare disease, there is a scarcity of randomized controlled trials of treatment. Consequently, there is a need for meta-analyses and systematic reviews to examine different treatment modalities. Here, we conducted such a meta-analysis based on a literature review.

## 2 Methods

**Figure 1:**
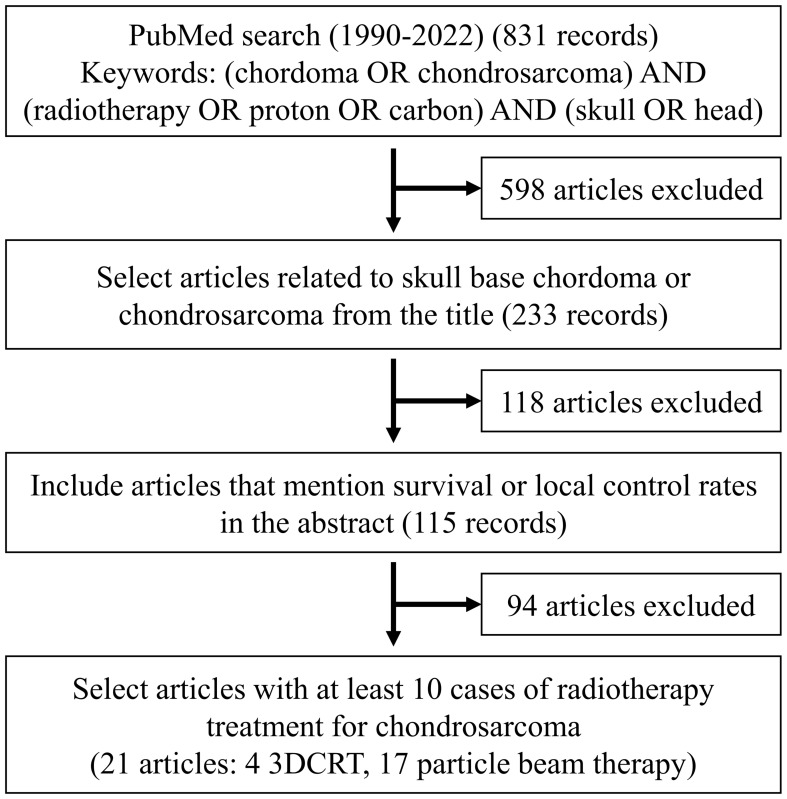
Process for selection of articles.

This review and meta-analysis were conducted following the guidelines and recommendations of the Preferred Reporting Items for Systematic Reviews and Meta-Analyses (PRISMA) (8, 9). A search was performed for relevant articles published in English between 1990 and 2022. The selection criteria are shown in **Figure 1**. The search was conducted in PubMed using the terms (chordoma OR chondrosarcoma) AND (radiotherapy OR proton OR carbon) AND (skull OR head). Among the 831 articles identified, two reviewers screened the titles to extract articles pertaining to skull base chordoma or skull base chondrosarcoma. In cases of uncertainty, a third reviewer was consulted for further evaluation. The reviewers examined the abstracts to identify those that specified survival or local control rates, resulting in the selection of 115 out of 233 abstracts. The full text of the selected abstracts was then read to extract information on variables such as the number of cases, survival rate, local control rate, gender, resection, resection rate, tumor size, tumor volume, prescription dose, number of fractions, and treatment modality. This process yielded a final selection of articles (4, 6, 10–24) with at least 10 cases per treatment modality, including 38 cases of chordoma and 21 cases of chondrosarcoma (**Table 1**). The extracted data comprised the author, year, sample size, country, article type, age, gender, tumor diameter, tumor volume, resection rate, and tissue type. Radiotherapy data, including the modality and prescribed dose, are shown in **Table 2**.

**Table 1:** List of selected articles.

Author	Year	Modality	Study	N	Volume (cc)	Male (%)	Age (year)	GTR (%)	BED10 (Gy(RBE))
Rimmer	2022	Proton	DB	60					
Patel	2021	Proton	DB	67					
Holtzman	2019	Proton	R	43	18	42	49		73
Simon	2018	Proton	R	23		57	42	0	70
Weber	2018	Proton	R	135					70
Mattke	2018	Proton	R	22	38	36	41	0	70
Weber	2016	Proton	R	71	36	44	36	4	70
Feuvret	2016	Proton	R	159	23	45	40	8	69
Weber	2016	Proton	R	77	25	45	39	0	70
Ares	2009	Proton	R	22		59		0	67
Weber	2005	Proton	R	11	15			0	68
Noël	2001	Proton	R	11	18	55	42	9	67
Hug	1999	Proton	R	25		36	44		69
Mattke	2018	Carbon	R	79	35	41	46	0	65
Riva	2021	Particle	R	48	14	40	50	6	70
Mattke	2018	Particle	R	101	38	40	44	0	
Castro	1994	Particle	R	27					
Rimmer	2022	3DCRT	DB	201					
Patel	2021	3DCRT	DB	218					
Noël	2004	3DCRT	R	26			36		67
Noël	2003	3DCRT	R	18	20	50	42	22	67

3DCRT, three-dimensional conformal radiation therapy; DB, treatment result from database; R, retrospective study; GTR, gross total resection; BED, biologically effective dose; RBE, relative biological effectiveness.

**Table 2:** Survival rates in the selected articles.

Author	Year	Follow-up period (months)	3y OS (%)	5y OS (%)	3y LC (%)	5y LC (%)
Rimmer	2022		98	95		
Patel	2021	128	97	97		
Holtzman	2019	44	95	95	93	
Simon	2018	91	100	100		
Weber	2018	88	93	93		
Mattke	2018	31	100		100	
Weber	2016	50				93
Feuvret	2016	77	93	92	98	96
Weber	2016	69		93	94	94
Ares	2009	34	91	91	94	94
Weber	2005	29			100	
Noël	2001	29	90		90	
Hug	1999	33	100	100	94	75
Mattke	2018	44				
Riva	2021	35			98	
Mattke	2018	40				
Castro	1994					78
Rimmer	2022			82		
Patel	2021	128		71		
Noël	2004	34	95		91	
Noël	2003	29	94		85	

OS, overall survival; LC, local control.

Random effects meta-analyses of 3- and 5-year overall survival (OS) and local control (LC) rates for each treatment modality were performed to generate forest plots. For studies with missing accuracy data, missing values were imputed using information on the number of cases, size of the risk set each year, and mean dropout rate. Heterogeneity in each meta-analysis was assessed by I-square statistics. Random-effects meta-regressions with modality as the explanatory variable were performed for each outcome to compare across modalities. All analyses were performed using R software (R Core Team, Vienna, Austria) and its meta package (25).

## 3 Results

**Figure 2:**
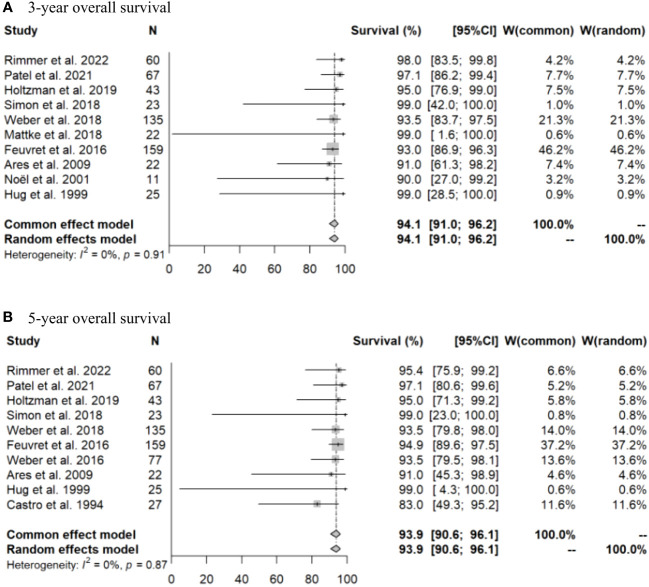
Forest plots for **(A)** 3-year and **(B)** 5-year overall survival rates after particle beam therapy.

**Figure 3:**
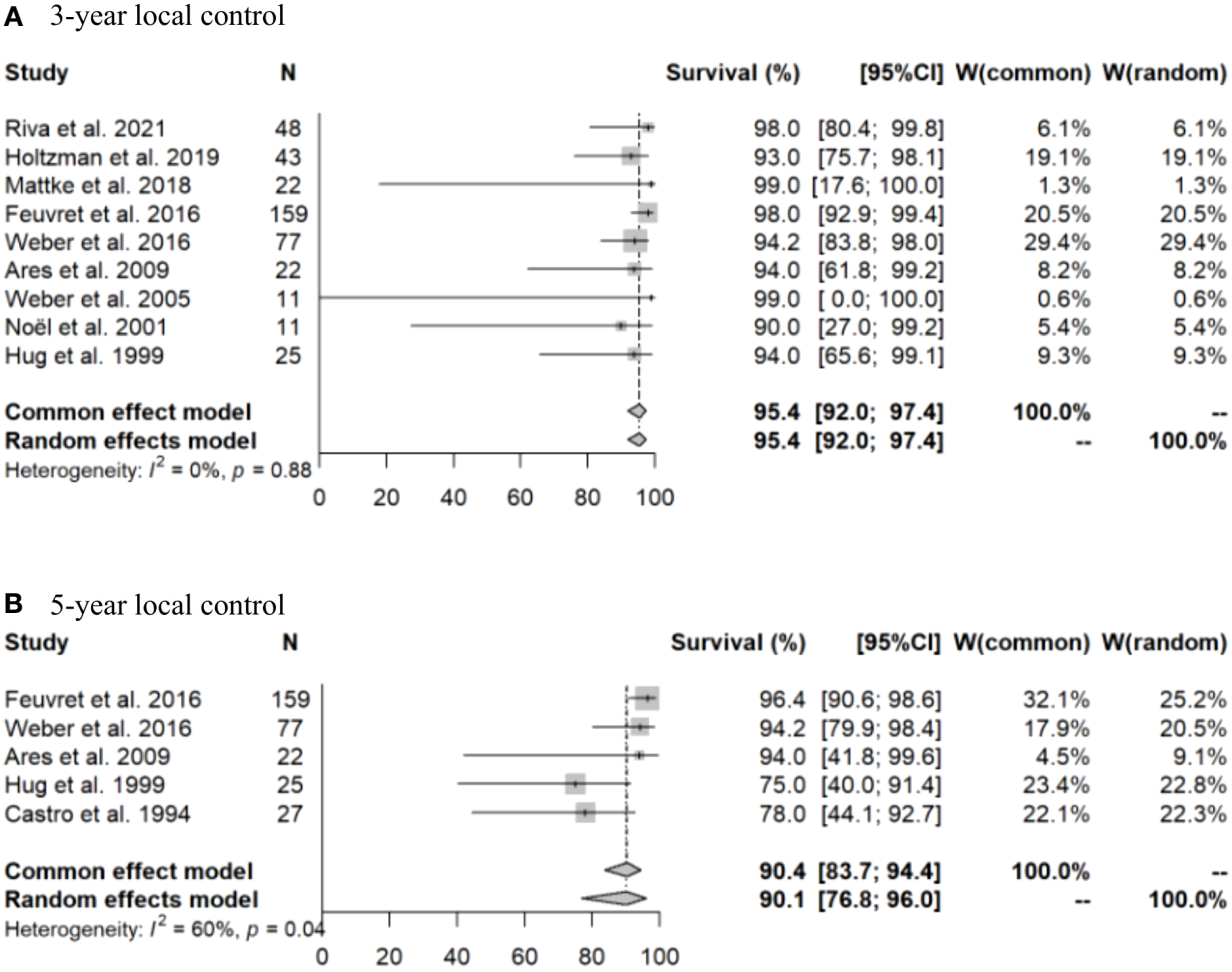
Forest plots for **(A)** 3-year and **(B)** 5-year local control rates after particle beam therapy.

Initially, a meta-analysis was conducted using all the selected literature. Due to the limited number of articles, it was challenging to generate forest plots for three-dimensional conformal radiotherapy (3DCRT). Therefore, forest plots for OS and LC rates are only presented for PT (**Figures 2**, **3**). After PT, the 3- and 5-year OS rates were 94.1% (95% confidence interval [CI]: 91.0-96.2%) and 93.9% (95% CI: 90.6-96.1%), respectively, and the 3- and 5-year LC rates were 95.4% (95% CI: 92.0-97.4%) and 90.1% (95% CI: 76.8-96.0%), respectively. Meta-regression analysis revealed a significant association between PT and a superior 5-year OS rate compared to 3DCRT (p < 0.001). Comparison of the 5-year LC rate for PT and 3DCRT could not be made due to insufficient data. The doses used in PT in the selected articles were 60 Gy (relative biological effectiveness [RBE]) in 20 fraction, 68 Gy (RBE) in 34 fraction, 70 Gy (RBE) in 35 fraction, 70.2 Gy (RBE) in 39 fraction, and 73.8 Gy (RBE) in 41 fraction. The major adverse events (AEs) of grade 2 or higher were temporal lobe necrosis (incidence 1-18%, median 7%) (6, 11, 13, 15, 21) and pituitary hypofunction (incidence 9-27%, median 16%) (6, 11, 15, 18).

## 4 Discussion

Chondrosarcoma of the skull base is a rare disease and is often reported together with chordoma of the skull base; however, several studies have suggested that chondrosarcoma has a better prognosis (26–29). Since these are locally invasive tumors, the best way to improve the local progression-free period is to remove as much tumor as possible while preserving function, with postoperative radiotherapy for cases of chondrosarcoma without complete resection (30, 31). The effects of different radiotherapy modalities have not been examined in this context, although the higher dose concentration as an advantage of PT over conventional 3DCRT has been noted (32, 33). This study was designed to examine this issue, but it was difficult to collect sufficient cases in the literature, and only a summary of the results of postoperative irradiation with PT was ultimately possible.

In this study, the 5-year OS was better after PT (93.9%) than after 3DCRT (71.0-82.3%) (p<0.001), which may reflect the value of postoperative PT. At our center, the 5-year LC rate after PBT for 18 cases of chondrosarcoma was 100%, and the prognosis for skull base chondrosarcoma was favorable (34). However, AEs from postoperative irradiation can be problematic. Advances in radiotherapy techniques have allowed delivery of higher doses to the target, but radiation brain necrosis, especially of the temporal lobe, is a major AE in irradiation of the skull base (6, 34, 35). Radiation brain necrosis is generally shown that the risk is significantly related to the dose and volume of irradiated normal brain (36, 37). In a report on stereotactic radiotherapy, the incidence of radiation brain necrosis was significantly higher when the volume of normal brain irradiated with >25 Gy exceeded 16cc or with >30 Gy exceeded 10 cc, when metastatic brain tumors were irradiated in 5 fractions (36). For conventional fractionation to partial brain, a 5% and 10% risk of symptomatic radiation necrosis is predicted to occur at 72 Gy [range, 60–84] and 90 Gy [range, 84–102], and the brain is especially sensitive to fraction sizes >2 Gy (37). In PBT, risk factors for temporal lobe brain necrosis include a volume of 5.5 cc irradiated at ≥60 Gy and a volume of 1.7 cc irradiated at ≥70 Gy (35). Our PBT facility has reported an incidence of brain necrosis of grade 2 or higher of 3.9% over 5 years, with the total dose at treatment associated with the development of brain necrosis (34). The incidence of temporal lobe necrosis of grade 2 or higher in the studies in the current analysis was 1-18% (median 7%), which is slightly higher than that at our center. The dose-volume-histogram for the temporal lobes in each study was a concern.

It was difficult to compare PBT vs, X-ray radiotherapy because of the small number of articles on X-ray. However, there are many references on PBT, and the results of a meta-analysis showed a good 5-year overall survival rate of 93.9% and a 5-year local control rate of 90.1%, so we think it was meaningful to show that PT is appropriate as postoperative irradiation for skull base chondrosarcoma. On the other hand, temporal lobe necrosis also occurred in 1-18% of patients, although the number of severe cases was small. The future challenge is to select an irradiation strategy that minimizes the probability of adverse events such as temporal lobe necrosis while maintaining this treatment outcome.

The limitation of this study is that it could not be performed in detail because of the lack of information on how to determine the margin to the target and the setting of the prophylactic irradiation range, and because of the different treatment policies at each center. The differences in PT due to different irradiation techniques (fixed beam or gantry-based beam and passive-scattering or scanning) were not discussed due to the lack of detailed information. Since the resection rate varies with time for surgery as well as radiotherapy, and the resection rate may affect the outcome of treatment, we tried to incorporate the resection rate as a risk factor, but the limitation is that we could not analyze it well because there are few articles specifying the resection rate. Moreover, it is not very accurate due to the small number of articles on X-ray radiotherapy, so the data will need to be updated in the future when more stereotactic radiotherapy and stereotactic radiosurgery articles are published.

## 5 Conclusion

Comparison of X-ray therapy with PT for skull base chondrosarcoma is challenging, but PT has a good outcome and may be a useful option among radiotherapy modalities. A good survival rate is likely after high-dose postoperative irradiation of skull base chondrosarcoma, but AEs such as temporal lobe necrosis may occur and there is room for improvement in both the choice of the radiotherapy modality and setting of the irradiation field.

## Data availability statement

The raw data supporting the conclusions of this article will be made available by the authors, without undue reservation.

## Author contributions

MN: Visualization, Writing – original draft, Writing – review & editing, Data curation. MM: Conceptualization, Data curation, Validation, Writing – original draft, Writing – review & editing. TS: Data curation, Writing – review & editing. SS: Data curation, Investigation, Writing – review & editing. YL: Data curation, Investigation, Writing – review & editing. YO: Data curation, Writing – review & editing. MI: Data curation, Writing – review & editing. SH: Conceptualization, Data curation, Writing – review & editing. HF: Conceptualization, Data curation, Writing – review & editing. RS: Conceptualization, Data curation, Writing – review & editing. TI: Conceptualization, Methodology, Writing – review & editing. KN: Conceptualization, Data curation, Writing – review & editing. KM: Formal analysis, Validation, Writing – review & editing. HS: Supervision, Writing – review & editing.
